# LINS, a modulator of the WNT signaling pathway, is involved in human cognition

**DOI:** 10.1186/1750-1172-8-87

**Published:** 2013-06-17

**Authors:** Nadia A Akawi, Fatma Al-Jasmi, Aisha M Al-Shamsi, Bassam R Ali, Lihadh Al-Gazali

**Affiliations:** 1 Department of Pathology, College of Medicine and Health Sciences United Arab Emirates University, P.O. Box 17666, Al-Ain, United Arab Emirates; 2 Department of Paediatrics College of Medicine and Health Sciences, United Arab Emirates University, P.O. Box 17666, Al-Ain, United Arab Emirates; 3 Department of Paediatrics, Tawam Hospital, Al-Ain, United Arab Emirates

## Abstract

**Background:**

Inherited intellectual disability (ID) conditions are a group of genetically heterogeneous disorders that lead to variable degrees of cognition deficits. It has been shown that inherited ID can be caused by mutations in over 100 different genes and there is evidence for the presence of as yet unidentified genes in a significant proportion of patients. We aimed at identifying the defective gene underlying an autosomal recessive ID in two sibs of an Emirati family.

**Methods:**

A combined approach involving homozygosity mapping and whole-exome sequencing was used to identify the causative mutation. RNA analysis was performed to gain further insight into the pathogenic effect of the detected mutation.

**Results:**

We have identified a homozygous splicing mutation (c.1219_1222+1delAAAGG) in the *LINS* gene in the affected children. *LINS* is the human homologue of the Drosophila segment polarity gene *lin* that encodes an essential regulator of the wingless/Wnt signaling. The identified mutation alters the first consensus nucleotide of the 5' donor splice junction of intron 5 and the 3' end of exon 5. Transcript analysis revealed that this change leads to an exon skipping event resulting in direct splicing of exon 4 to exon 6. Another mutation in *LINS* has been described very briefly in an Iranian family with autosomal recessive ID and microcephaly.

**Conclusion:**

Our study confirms that *LINS*, a modulator of the WNT pathway, is an indispensable gene to human cognition and this finding sheds further light on the importance of WNT signaling in human brain development and/or function.

## Introduction

Intellectual disability (ID) is a health condition characterized by low intelligence and associated limitations in adaptive behavior. ID is a highly heterogeneous condition and one of the most important socio-economic health care problems worldwide [1]. Molecular karyotyping is the first diagnostic test for congenital ID as most severe cases occur due to chromosomal abnormalities [2]. High resolution comparative genomic hybridization (CGH) was developed to detect pathogenetically relevant deletions and duplications too small to be detectable by conventional karyotyping [3]. Sequencing, on the other hand, has become the method of choice to diagnose causes of ID that cannot be explained by routine karyotyping or CGH [4]. During the past decade, hundreds of defective genes have been identified to be the underlying causes of ID [5]. Different modes of Mendelian inheritance have been reported to cause ID with the vast majority of cases are inherited as an autosomal recessive trait [2].

Several autosomal recessive ID genes in families from the United Arab Emirates (UAE) have been identified using the concept of homozygosity mapping and candidate gene approach [6-8], and more recently using both homozygosity mapping and exome sequencing [9-12]. In 2011, a collaborative study was carried out on consanguineous Iranian families with autosomal recessive ID [13]. The authors combined homozygosity mapping and exome sequencing to unravel the molecular basis of ID in many families. This study has revealed new mutations in 23 genes previously implicated in autosomal recessive ID, and disease causing variants in 50 novel genes including *LINS* (OMIM#610350). However, very limited information has been provided on the patients’ phenotype and the implications of the reported mutation. Here, we report two siblings, a male and a female with early onset ID, harboring a novel five nucleotide homozygous deletion in *LINS* gene. The mutation affects a donor splice site leading to exon skipping and a large deletion in the expressed transcripts.

In Drosophila, lines is the homologue of LINS and has been recognized to be a tissue- and a stage-specific modulator of wingless signaling [14]. Lines was found to be activated by Drosophila wingless (wg) [14]. Wingless-type MMTV integration site family-1 (WNT1) is the human homologue of the Drosophila wg and its discovery led to the subsequent elucidation of the WNT pathway [15]. The activation of the canonical wingless/WNT signaling pathway occurs through the binding of wg/WNT ligand to the seven-pass transmembrane Frizzled (Fz) receptor and its co-receptor, the arrow (arr)/low-density lipoprotein receptor related protein (LRP) [16]. This binding stabilizes the cytosolic co-activator armadillo (arm)/β-catenin1(CTNNB1) and its translocation to the nucleus [15]. Thus, leading to competitive displacement of groucho (gro)/transducin-like enhancer of split (TLE) from the transcription factors pangolin (pan)/T cell-specific transcription factor (TCF) initiating the transcription of the pathway target genes. WNT1 is secreted from a signaling center located at the boundary between prospective mid and hindbrain (mid-hindbrain boundary) and mediate development of these two brain regions [17]. Disturbed WNT pathway due to inherited mutations in positive and negative regulators of the signaling have been reported to cause autosomal recessive ID [18,19]. Therefore, our finding that a mutation in another regulator of the WNT signaling pathway is responsible for a form of recessive ID further illustrates the importance of this pathway in human cognition and/or brain development.

## Materials and methods

### Research subjects

**Figure 1:**
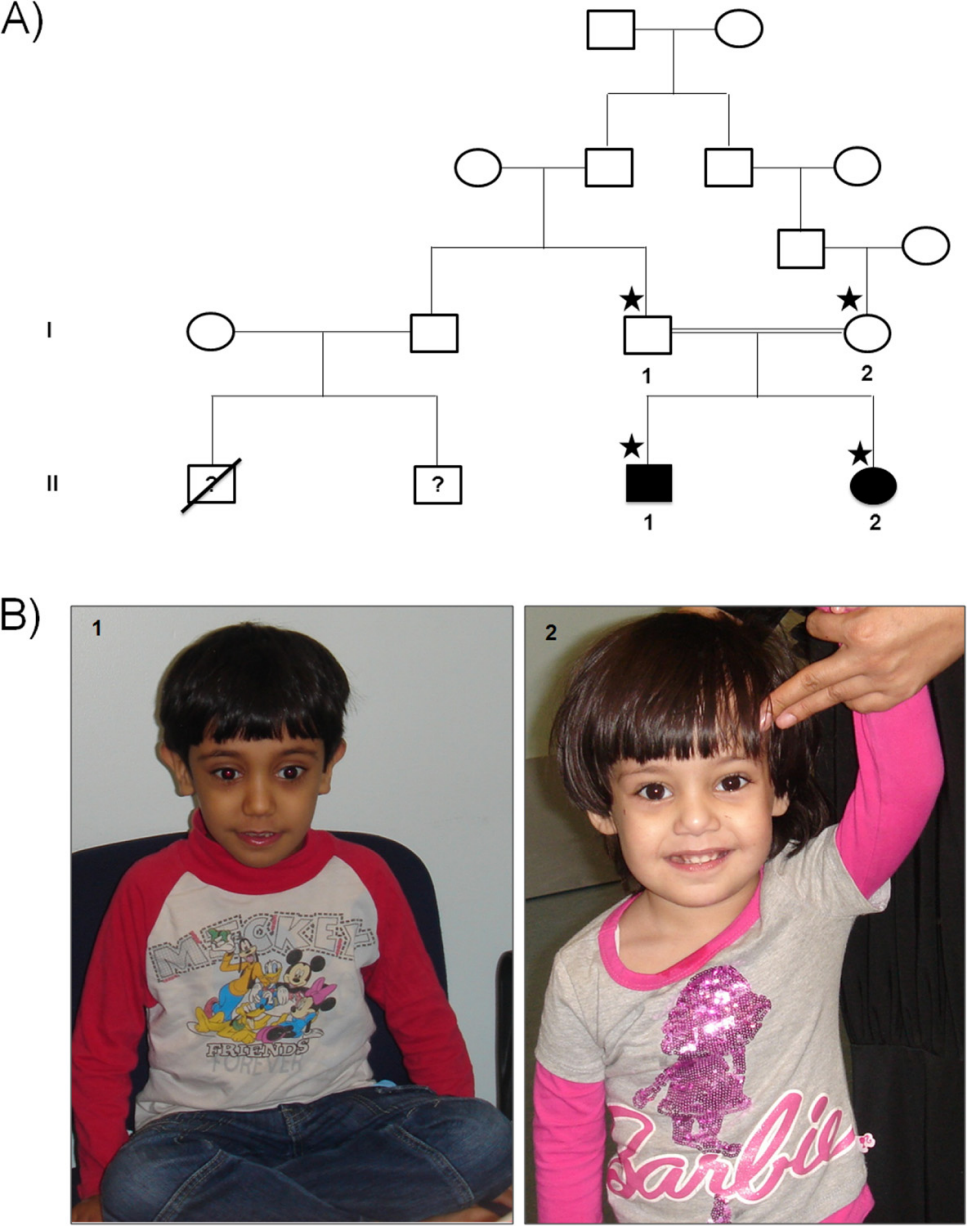
**Clinical Data of the studied family. A**. Pedigree showing the mode of inheritance for an autosomal recessive intellectual disability phenotype in a consanguineous family from United Arab Emirates. The studied members are indicated by numbers and asterisks. **B**. Appearance of the 2 affected sibs, 1) II1 at age 8 years and 2) II2 at age 3 years. Note the flattening of the midface.

One consanguineous family with two affected children exhibiting an early onset ID was recruited for this study (Figure 1). The study was approved by Al-Ain District Human Research Ethics Committees and the family provided a written informed consent for participating in the study.

### Clinical report

The parents of the two affected children are Emirati first cousins once removed of Yemeni origin (Figure 1A). They have 2 children; both of them are affected by intellectual disability. In the family history the father’s brother had a child who died at 6 months of age of unknown cause and a 14 year old child with intellectual disability of unknown etiology. No further information was available on this child and we were unable to evaluate her because she lives in Yemen.

The first child of this family is a boy and currently aged 9 years (Figure 1A-II1). The pregnancy was complicated by gestational diabetes and mild hypertension, delivery was induced but otherwise was normal. His birth weight was 3000 gm but no other measurements were available. The neonatal period was complicated by poor feeding requiring admission to the Special Baby Care Unit (SCABU) for several days. At the age of 9 months he was not responding to the mother and was noted to have head nodding and repetitive rotatory hand movements. The hand movements disappeared but the head nodding continued till now. He crawled at the age of 16 months and walked at the age 19 months but he still has no speech. He was extremely hyperactive with aggressive destructive behavior for which he required medications to calm him down. There was no history of seizures. Examination at the age of 8 years revealed a weight of 18 kg (<3^rd^ centile) and height of 105 cm (<3^rd^ centile), and head circumference of 51 cm (<50^th^ centile). He had slightly flat midface with depressed nasal bridge (Figure 1B-1) otherwise no other dysmorphic features were noted. He was continuously nodding his head from side to side. Neurological examination was normal. EEG and skeletal examinations were reported to be normal. MRI brain showed right frontal lobe vascular malformation with cortical and subcortical distribution. No associated cortical abnormalities were observed. No hemorrhage or gliosis and MRI Spectrometry was normal. Blood and urine amino acid and organic acid screening, thyroid function tests, mucopolysaccharides screening, transferring isoelectric focusing, very long chain fatty acids and phytanic acids, Fragile X mutation, *MECP2* gene analysis were all normal. CGH microarray analysis was normal.

**Figure 2:**
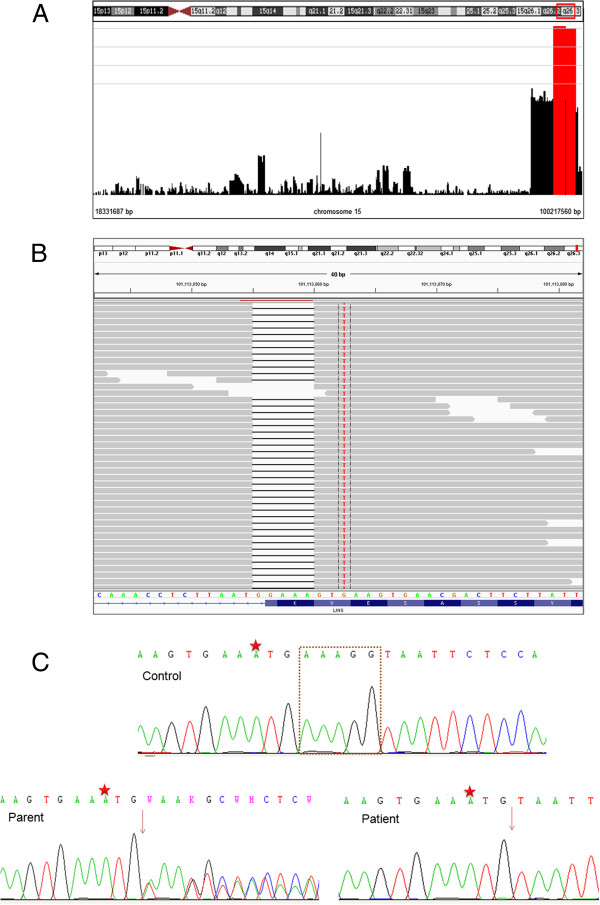
**Genomic mapping and genetic testing of an autosomal recessive intellectual disability phenotype. A**. Genotyping the whole genome of both parents (I1 and I2) and affected children (II1 and II2) detected a homozygosity on chromosome 15q26. **B**. Integrative Genomics Viewer (IGV) visualization of homozygous mutation c.1219_1222+1delAAAGG in *LINS* gene from exome data. All reads show 5 bp deletion, sequence of wild type gene and exon annotation at bottom. The adjacent homozygous substitution G>A (C>T on reverse strand) is a common variant rs12719734G>A. **C**. DNA sequencing chromatograms confirmed the segregation of the AAAGG (inside the brown square) deletion detected by exome data with the assessed phenotype. The deletion was found to be homozgous in the patients (II1 and II2) and heterozygous in parents (I1 and I2). The deletion was not found in 100 normal controls. The rs12719734G>A (designated with a red star) was found in all the screened individuals.

The second child is a 3 years old female (Figure 1A-II2; Figure 1B-2). She is the product of normal pregnancy and delivery. Her birth weight was 2950 gm. The mother noted head nodding in the first few months of life. She was hypotonic and had head lag at the age of 7 months. All developmental milestones were delayed. She walked at 20 months of age and she has no speech till now. Examination at 7 months revealed mild flattening of midface. No other dysmorphic features were noted. Neurological examination revealed hypotonia with head lag, side to side head nodding, otherwise no other abnormalities. EEG showed bilateral centro-temporal discharge without generalization. MRI brain was normal. Creatine phosphokinase (CPK), uric acid, lactate, urine and blood amino acids and organic acids were normal. Transferrin-isoelectric focusing and very long chain fatty acids and phytanic acids were normal. CGH microarray showed interstitial deletion of 4 oligonucleotide probes at 7p22.1 spanning approximately 197 kb. However, testing the parents showed that the mother has these changes and the other affected child did not have them indicating that this deletion is not related to the phenotype. At 3 years her weight was 13 kg (3^rd^ centile), height 92 cm (3^rd^ centile) and head circumference 48 cm (3^rd^ centile) (Figure 1B-2).

### DNA extraction

Genomic DNA was isolated from blood collected in EDTA tubes from all the family members (parents and affected children) using flexigene DNA extraction kit (Qiagen GmbH, Hilden, Germany).

### Genotyping and linkage analysis

Genotyping of the whole genome of the studied individuals in this family was undertaken using GeneChip Genome-Wide Human SNP Array 6.0 (Affymetrix, Santa Clara, CA, USA). SNP genotypes were obtained by following the standard protocol supplied by the manufacturer. Genotypes were called with the Genotype Console program (Affymetrix, Santa Clara, CA, USA). Generated SNPs derived from the family members' DNA were loaded into the software package HomozygosityMapper [20,21] and subjected to computational linkage analysis assuming a fully penetrant autosomal recessive mode of inheritance.

### Whole-exome sequencing and bioinformatics analysis

Sequencing library construction, exome capture, sequencing, and standard data analyses for the affected children in this family was performed by Oxford Gene Technology (Oxfordshire, UK). Exome capturing and enrichment was carried out using SureSelect All Exon V4 kit (Agilent Technologies, Santa Clara, CA, USA) following the manufacturers' protocols. Whole exome sequencing was carried out on Illumina HiSeq 2000 system (Illumina, San Diego, CA, USA). Paired end (2×100 bases) DNA sequence reads that passed the quality control were mapped to the human reference genome build hg19 using the BWA [22] and SAM tools [23]. All the annotated variants were filtered against dbSNP, 1000Genome project, NHLBI exome sequencing project and in-house exome variants databases. SIFT [24], Polyphen2 [25], and MutationTaster [26] prediction programs were used to predict the impact of each variant on the structure and function of the protein product.

### Transcript analysis

Total RNA was isolated from fresh blood with QIAamp RNA blood kit (Qiagen GmbH, Hilden, Germany), and single-stranded cDNA was synthesized with the GoScript reverse transcription system in accordance with the manufacturer’s instructions (Promega, Madison, USA). To investigate the effect of the detected splice site mutation and to avoid genomic amplification, RT-PCR was carried out with primers spanning the exon–exon junctions of NM_001040616.2. This transcript is the only validated isoform that encodes a functional protein (NP_001035706.1; 757aa) encompassing 7 exons where exon 1 is non-coding [27]. The forward primer (5’-CGATTCTAAATTAATCTGCATGTTCC-3’) spans the junction between exons 2 and 3 while the revers primer (5’-CATCCTCTGGTCAGTGTTAAG-3’) spans the junction between exons 6 and 7. The PCR products were separated on a 2% agarose gel. The relevant bands were purified from gel using MinElute gel extraction kit (Qiagen GmbH, Hilden, Germany), and sequenced using Sanger sequencing.

### Sanger DNA sequencing

Direct DNA and cDNA sequencing was carried out using the BigDye Terminator kit v3.1 (Applied Biosystems, Foster, CA, USA). Purified PCR amplification products and gel purified bands were sequenced using the DNA sequencing with fluorescent automated sequencing on the ABI 3130*xl* genetic analyzer (Applied Biosystems, Foster, CA, USA).

## Results and discussion

### Results

#### Genome-wide linkage analysis revealed four homozygous regions

The results of the genome-wide SNP genotyping and linkage analysis for the studied pedigree are shown in Figure 2A, Additional file 1: Table S1, and Additional file 1: Figure S1. The genotyping data showed four blocks of homozygosity shared between the two affected members of the studied family (Additional file 1: Figure S1A). One block mapped to chromosome 8 between rs7388114 and rs4738955 flanking a 2.5 Mb genetic interval (8q12.1-q12.3) (Additional file 1: Figure S1B). The second block of homozygosity mapped to chromosome 10 between rs293303 and rs10994485 flanking a 9.2 Mb genetic interval (10q21.1-q21.2) (Additional file 1: Figure S1C). Blocks of homozygosity were observed on chromosome 13, which together comprised 19.9 Mb genetic interval (13q21.2-q33.3) (Additional file 1: Figure S1D, Table S1). The last block of homozygosity detected was found on chromosome 15 spanning 3.8 Mb between rs1588752 and rs11637451 (Figure 2A). The parents were heterozygous at all the homozygous segments.

#### Whole-exome sequencing identified a splicing mutation in LINS gene

The co-segregating homozygous segments together encompass around 163 genes (Additional file 1: Table S1). In order to reveal the molecular basis of the ID in the studied family, whole-exome sequencing was carried out on the two affected children. A minimum of 79.70% of the on-target regions were covered to a depth of at least 20x. Around 45,800 variations from the reference genome were identified (Additional file 1: Table S2). Among these 3,500 novel variants were recognized and approximately 700 variations indicative of serious consequences in coding sequences were found. Across the variations, 160 variants were found to be homozygous, of which only two were shared between the two affected children. Both variants were within the same homozygous region on chromosome 15q26. Both were splicing mutations affecting a splice donor in *LINS* (NM_001040616.2: c.1219_1222+1delAAAGG) and a splice acceptor in *TTC23* (NM_001040655.1:c.456-1G>T) (Figure 2B, Additional file 1: Figure S2). Both variants were confirmed to be homozygous in the two affected children, heterozygous in parents and not found in 200 healthy controls with matching ethnic origin by Sanger sequencing (Figure 2C). However, *LINS* has been concluded to be the causative gene because it has been recently linked to autosomal recessive ID in an Iranian family [13].

#### The c.1219_1222+1delAAAGG mutation in LINS gene caused Exon 5 skipping

**Figure 3:**
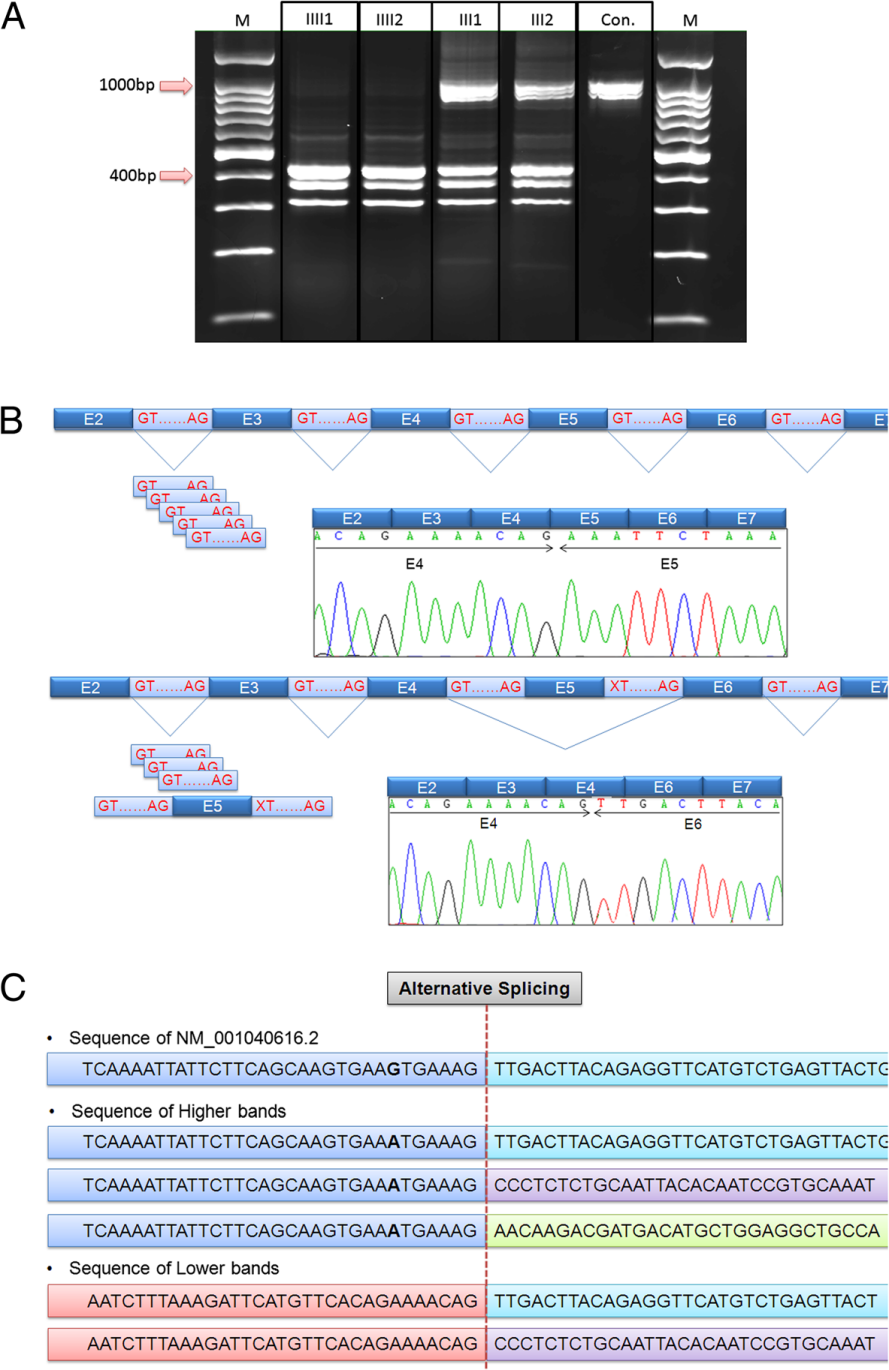
**Exon skipping was assessed by reverse transcription-PCR and Sanger sequencing. A**. Agarose gel of RT-PCR reaction products from *LINS* cDNA amplification in a control (con), parents (I1, I2) and patients (II1, II2) compared to a DNA 100bp ladder (M). The gel showed a ~1014bp band of the wild type *LINS* transcript encompassing exon 5 in a normal control (con) accompanied with multiple isoforms of varying length (~1000bp). In patients (II1, II2) lanes, only smaller bands were seen (~400bp) suggesting a homozygous deletion of around 600bp. The parents (-I1, -I2) have both upper and lower bands suggesting that they carry the 600bp deletion in a heterozygous state. **B**. A schematic diagram of the splicing defect seen in patients based on Sanger sequencing data of the cDNA. The upper most band of the higher and lower bands seen in RT-PCR gel were purified and sequenced. This higher band noticed in control and parents was found to include exons 3,4,5 and 6. On the other hand, Exon 5 (E5) was found to be missing in the lower-size band seen in both patients and parents. These results suggested that the genomic deletion at the end of E5 abolished a canonical splicing site masking the exon from the splicing machinery which considered it to be part of intron 4 and cut it out of the nascent mRNA. **C**. Analyzing the accompanying upper and lower bands amplified by RT-PCR suggested the presence of at least 3 *LINS* transcripts alternatively spliced in exon 6 with the putative multiple splice junctions are shown. All the lower bands lack exon 5 while the upper bands include it compared to the RefSeq NM_001040616.2.

To investigate the consequences of the molecular defect caused by the detected splicing mutation, RT-PCR was performed using total RNA isolated from a normal control, parents and patients’ leukocytes as templates (Figure 3A). The control sample (Con.; Figure 3A) showed multiple bands at around 1000bp indicating the presence of multiple transcripts for this gene in leukocytes. On the other hand, the two patients showed similar multiple bands pattern, albeit at lower sizes of around 400bp (Figure 3A-II1 and -II2). The parents showed both the upper and the lower multiple bands which is consistent with being heterozygous carriers for the predictable splicing aberration (Figure 3A-I1 and -I2).

To further characterize the spliced products, we gel-purified all the PCR bands and sequenced them using Sanger sequencing. The analysis demonstrated that in the normal control the upper band (1014bp) represented the NM_001040616.2 cDNA fragment spanning from exon 3 to exon 6 (Figure 3B). Interestingly, the higher band was accompanied by at least two bands recognized to be alternatively spliced transcripts which lacked some parts of exon 6 (Figure 3C). The exon-intron 5 splice defect mutation present in the patients’ gene caused the skipping of exon 5 resulting in a smaller sized band (423bp) noted in the parents and patients but not in the normal control (Figure 3B). This was also accompanied by bands of lower sizes representing multiple transcripts for the mutated allele (Figure 3C). As indicated above, these additional splice variants that lack parts of exon 6 are also present in the control DNA and therefore not related to the pathogenic phenotype.

#### Bioinformatic analysis predicted that exon 5 skipping is deleterious to the corresponding protein

Katoh [27] characterized human LINS (NP_001035706.1) and mouse Lins by their similarity with Drosophila lines. The two proteins shared a homologous domain with Drosophila lines with the human protein consisting of 757 amino acids (aa). Translating NM_001040616.2 lacking exon 5 by Expasy translate tool [28] predicted a truncated protein lacking 197 amino acid (p.Glu211_Lys407del). Most of these deleted amino acids are evolutionarily conserved across species suggesting an important role for this domain in the protein structure and/or function (Additional file 1: Figure S3). Part of the deletion (30aa) lies within the Drosophila lines homologous domain found by Katoh [27]. The deletion also included Lys407 which is found experimentally to be a potential regulator of the protein ubiquitination and the subsequent regulation of its proteasome-mediated degradation [29].

## Discussion

Numerous studies have revealed that correct corticogenesis is an outcome of the interplay between multiple signaling pathways including Wg/WNT, Hedgehog (Hh) and Notch (N) pathways [30-36]. This crosstalk provides mitogenic signals, positional information, migratory cues and differentiation signals [33]. In addition, the coordinated interaction between these critical pathways is a prerequisite for the precise regulation of symmetric/asymmetric division during neurogenesis in the developing vertebrate central nervous system (CNS). Many of these pathways were first identified in genetic studies in Drosophila [34]. Mammalian orthologs were subsequently identified and genes within the pathways have been cloned and studied. However, the exact outcomes of these interactions are not fully understood. In addition, not all the interactive players or factors that affect the number and type of divisions that a neocortical progenitor cell undergoes are known.

**Figure 4:**
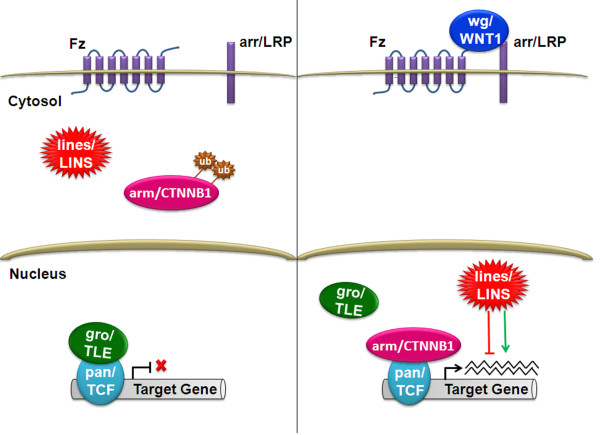
**Lines/LINS plays a putative dual role in WNT canonical pathway.** In WNT canonical pathway, the absence of a signal leads to the hyperphosphorylation of arm/CTNNB1 leading to its ubiqitination and degradation by the proteasome in the cytoplasm. Binding of wg/WNT1 ligand to a Fz and arr/LRP receptor complex leads to stabilization of hypophosphorylated arm/CTNNB1, translocating it to the nucleus. In the nucleus, arm/CTNNB1 competes with and displaces gro/TLE interacting with pan/TCF proteins to activate transcription. In Drosophila, lines/LINS was found to act as a modulator of wg/WNT canonical pathway acting in parallel with or downstream of arm/CTNNB1 in response to wg/WNT1 signaling to enhance or represses the transcription of target genes. Frizzled (Fz), arrow (arr), LDL receptor-related protein (LRP), armallido (Arm), β-catenin (CTNNB1), groucho (gro), transducin-like enhancer of split (TLE), pangolin (pan), T-cell factor (TCF).

*LINS* (formerly known as *WINS1* or *LINS1*) is the human homologue of the Drosophila segment polarity gene *lin*[27]. Lines is the protein product of *lin* which was originally identified in Drosophila melanogaster in the 1980s [37,38]. Drosophila studies revealed that lines is an essential protein for patterning and morphogenesis of Drosophila dorsal epidermis [14,39,40], hindgut [41-43] and muscles [44]. Lines was also found to play an important role in the development of Drosophila wings [45,46], and testis [47]. Lines is believed to be a transcriptional regulator, playing a dual role as both an activator and repressor of downstream target genes listed in Table 1[14,41]. Hatini et al. [14] demonstrated that lines is essential for late wg signaling activity in the developing dorsal epidermis, acting downstream of arm but upstream of wg target genes (Figure 4). The author showed that with wg signaling, lines accumulates in the nucleus to modulate transcription of *wg* and *ve* (veinless) that are known targets of wg signaling. The author also proved that there is an interaction between lines and Drosophila hedgehog (hh) which exports lines from the nucleus to the cytoplasm antagonizing wg signaling. During Drosophila embryogenesis, lines found to be implicated in dorsal muscle patterning by regulating groovin expression [44]. In the developing hindgut, Iwaki et al. [41] demonstrated that lines promotes the expression of genes of the large intestine (*otp, dpp, en,* and *dri*), and represses the expression of genes of small intestine (*hh, upd,* and *Ser*). Castelli-Gair [48] proposed lines to be a transcriptional cofactor for Abdominal-B for the activation or repression of its downstream target genes. These genes include *cut* that represses a neural cell fate, *spalt* that affects the development of the fly's gut, and *ems* which is necessary for proper head formation and is also involved in brain morphogenesis [48,49]. It was shown that lines is part of a molecular regulatory pathway composed of drm, an inhibitor of lines by exporting it to the cytoplasm, and bowl a downstream target of lines in the nucleus [40]. Interestingly it was observed that, hh promotes *drm* expression, while wg represses *drm* expression regulating the drm/lines/bowl pathway which consequently regulates the patterning and cell rearrangement in the Drosophila embryonic epidermis, foregut, hindgut, gonads and imaginal disc [40,41,45,47]. In the developing wing, Benítez et al. [46] noticed that bowl protein represses Wg pathway and activates Notch (N) and Hh pathways. Therefore, they concluded that lines is essential for normal functioning of Wg, Hh and N pathways during embryogenesis in Drosophila. In the Drosophila testis, *lin* mutant cells were not differentiating into cyst stem cells (CySC) and expressed niche cell fate markers hh and cactus [40]. The observation suggested that lines represses niche fate and promotes CySC fate antagonizing Bowl and N pathway which promotes niche cell fate.

**Table 1:** The reported downstream target genes of lines in Drosophila

**Gene name**	**Gene symbol**	**Tissue/System**	**Up/Down**	**Human homologue**	**Putative roles in the development of the central nervous system in vertebrates**	**References**
wingless	*wg*	Dorsal Epidermis	Up	*WNT1*	WNT1 protein involved in the proliferation and differentiation of neural progenitors. Wnt1 deficient mice embryos have showed severe abnormalities in the development of the midbrain and cerebellum.	[14,35,50]
rhomboid	*rho (ve)*	Dorsal Epidermis	Down	*PARL*	This gene encodes a mitochondrial integral membrane protein that plays an important regulatory role in mitochondrial-mediated apoptosis. *Parl* knockout mice undergo progressive multi-tissue atrophy, including atrophy in the thalamus and striatum, mediated by increased apoptosis.	[14,51,52]
orthopedia	*otp*	Hindgut	Up	*OTP*	This gene encodes a homeodomain-containing transcription factor that is implicated in the development of the brain, specifically hypothalamus, in vertebrates. *Otp* knockout mice displayed progressive impairment of crucial neuroendocrine developmental events.	[41,53-55]
decapentaplegic	*dpp*	Hindgut	Up	*SMAD3*	This protein functions as a transcriptional modulator thought to play a role in the in neural stem cells where it is essential to activate TGFβ-responsive genes activating the neural developmental program.	[41,56]
engrailed	*en*	Hindgut/Posterior spiracles	Up	*EN1 and EN2*	Both genes encode homeodomain-containing transcription factors that have been implicated in the control of mid-hindbrain pattern formation during embryogenesis. En1 deficient mice lack most of the cerebellum and midbrain, whereas *En2* mutants survive with cerebellar defects.	[41,57,58]
retained	*retn (dri)*	Hindgut	Up	*-*		[41]
hedgehog	*hh*	Hindgut	Down	*SHH*	This gene encodes a protein that is crucial in patterning and cell-fate specification, particularly in the central nervous system. SHH plays different roles depending on its concentration, area, and timing of exposure.	[41,59]
outstretched	*os (upd)*	Hindgut	Down	*-*		[41]
Serrate	*ser*	Hindgut	Down	*JAG2*	The encoded protein is one of several ligands that activate Notch and related receptors. It was found in most neuron subtypes. Notch signaling plays a pivotal role in the regulation of vertebrate neurogenesis and brain development.	[41,60-62]
Brother of odd with entrails limited	*bowl*	Dorsal epidermis/foregut/hindgut/gonads/imaginal disc	Up	-		[40]
Cut	*ct*	Posterior spiracles	Up	-		[48]
Spalt major	*salm*	Posterior spiracles	Up	-		[48]
empty spiracles	*ems*	Posterior spiracles	Up	*EMX2*	The encoded protein is expressed in the dorsal telencephalon during development and is involved in regional patterning of the neocortex into defined functional areas. Emx2 deficient mice displayed defects in archipallium structures that are believed to play essential roles in learning, memory and behavior.	[48,63,64]
Stripe or Groovin	*sr*	Dorsal epidermis/muscle	Up	-		[44]
Cactus	*cact*	Testis	Down	*NFKBIA*	This gene encodes a member of the nuclear factor-κB (NF-κB) inhibitor family that is involved in inflammatory responses. NF-κB pathway plays a significant role in neurite outgrowth, activity-dependent plasticity, and cognitive function. NFKBIA is often deleted in glioblastomas.	[47,65,66]

In humans, LINS was described in 2002 by Katoh as a protein containing Drosophila lines homologous domain [27]. The author detected *LINS* 2.8 kb-transcript (NM_001040616.2) in human fetal brain and kidney. However, since then not many experiments were performed to characterize human LINS further. However, it has been recently suggested as a disease causing candidate for an autosomal recessive ID phenotype [13]. The authors identified a homozygous deletion of four nucleotides in *LINS* exon 5 (NM_001040616.2:c.985_988delCATG). This deletion was predicted to cause a frame shift producing a truncated protein (p.His329*). The mutation was found in four affected children of consanguineous parents exhibiting microcephaly and early onset ID. Our patients had no microcephaly but showed ID and head nodding as the only clinical features. The two families share ID and somehow similar destructive mutations confirming the importance of LINS in the cognitive pathways. Further experiments are needed to gain further insight into the pathogenic role of the *LINS* gene in brain and CNS dysfunction.

## Competing interests

All authors have non-financial interests that may be relevant to the submitted work.

## Authors’ contributions

LA and BA initiated, planned and coordinated the study. NA performed the bioinformatics interpretation of the genotyping and exome sequencing data and carried out the functional studies. LA recruited and diagnosed the family described herein and collected the clinical data. BA and LA oversaw all aspects of the research. LA, BA and NA wrote the manuscript. AS and FA contributed to the diagnosis and recruitment of the family. All authors read, edited and approved the final version of the manuscript.

## Supplementary Material

Additional file 1: Table S1. Intervals of shared homozygosity between the two affected individuals of the studied family. **Table S2.** Summary metrics of all and novel variants identified by the exome sequencing. **Figure S1.** HomozygosityMapper view of the identified homozygous regions in the studied family. **Figure S2.** IGV view of the second homozygous mutation detected by whole exome sequencing on Chr15:99758919C>A in *TTC23* gene. **Figure S3.** Conservation across species of the amino acids that are predicted to be deleted from LINS protein in the patients. Click here for file
