# Supplementary material for: LINS, a modulator of the WNT signaling pathway, is involved in human cognition
Source: Orphanet J Rare Dis. 2013 Jun 17;8:87. doi: 10.1186/1750-1172-8-87 (PMC3847167; doi:10.1186/1750-1172-8-87)
Supplement: Additional file 1: Table S1. — Intervals of shared homozygosity between the two affected individuals of the studied family. Table S2. Summary metrics of all and novel variants identified by the exome sequencing. Figure S1. HomozygosityMapper view of the identified homozygous regions in the studied family. Figure S2. IGV view of the second homozygous mutation detected by whole exome sequencing on Chr15:99758919C>A in TTC23 gene. Figure S3. Conservation across species of the amino acids that are predicted to be deleted from LINS protein in the patients. [file 1750-1172-8-87-S1.doc]

**Supplementary Table S1.** Intervals of shared homozygosity between the two affected individuals of the studied family.

| **Chr** | **Start** | **End** | **SNP start** | **SNP end** | **Genetic interval** | **Length (kb)** | **Genes** |
| --- | --- | --- | --- | --- | --- | --- | --- |
| 8 | 60,741,289 | 63,260,182 | rs7388114 | rs4738955 | q12.1-q12.3 | 2,518,893 | 17 |
| 10 | 53,237,721 | 62,487,332 | rs293303 | rs10994485 | q21.1-q21.2 | 9,249,611 | 33 |
| 13 | 61,358,370 | 74,891,328 | rs3119852 | rs9543569 | q21.2-q22.1 | 13,532,958 | 89 |
|  | 100,588,543 | 105,390,934 | rs7994515 | rs773354 | q32.3-q33.2 | 4,802,391 |  |
|  | 107,638,292 | 109,171,572 | rs11616550 | rs11618877 | q33.3 | 1,533,280 |  |
| 15 | 97,559,785 | 101,322,542 | rs1588752 | rs11637451 | q26.2-q26.3 | 3,762,757 | 24 |
| Total |  |  |  |  |  | 35,399,890 | 163 |

**Supplementary Table S2.** Summary metrics of all and novel variants identified by the exome sequencing

|  | **All Variants^*^** | | **Novel Variants**^¥^ | |
| --- | --- | --- | --- | --- |
|  | **II1** | **II2** | **II1** | **II2** |
| **Number of Variations** | 45,912 | 45,795 | 3,658 | 3,424 |
| **Variations Overlapping Genes** | 45,565 | 45,476 | 3,596 | 3,365 |
| **Variations Overlapping Transcripts** | 45,565 | 45,476 | 3,596 | 3,365 |
| **Variations Overlapping Regulatory Regions** | 9,314 | 9,223 | 876 | 804 |
| **Variations Overlapping Protein Domains** | 30,375 | 30,524 | 2,425 | 2,261 |
| **Intergenic Variations** | 347 | 319 | 62 | 59 |
| **Variations With Predicted Serious Consequences** | 10,050 | 9,940 | 793 | 680 |
| **Variations With Other Predicted Consequences** | 43,154 | 43,039 | 3,418 | 3,211 |
| **Homozygous Variations With Predicted Serious Consequences** | 8122 | 8096 | 165 | 160 |

^*^including those in dbSNP release 132.

^¥^filtering out those contained in dbSNP release 132.

|  |  |  |  |  |  |  |  |
| --- | --- | --- | --- | --- | --- | --- | --- |
|  |  |  |  |  |  |  |  |
|  |  |  |  |  |  |  |  |
|  |  |  |  |  |  |  |  |
|  |  |  |  |  |  |  |  |
|  |  |  |  |  |  |  |  |
|  |  |  |  |  |  |  |  |
|  |  |  |  |  |  |  |  |
|  |  |  |  |  |  |  |  |
|  |  |  |  |  |  |  |  |
|  |  |  |  |  |  |  |  |
|  |  |  |  |  |  |  |  |


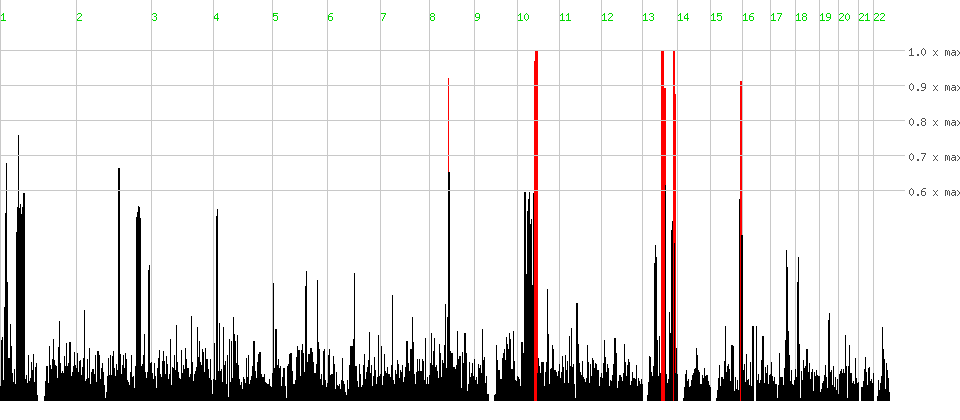
**A)**

**B)**


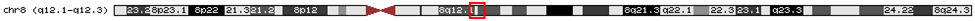

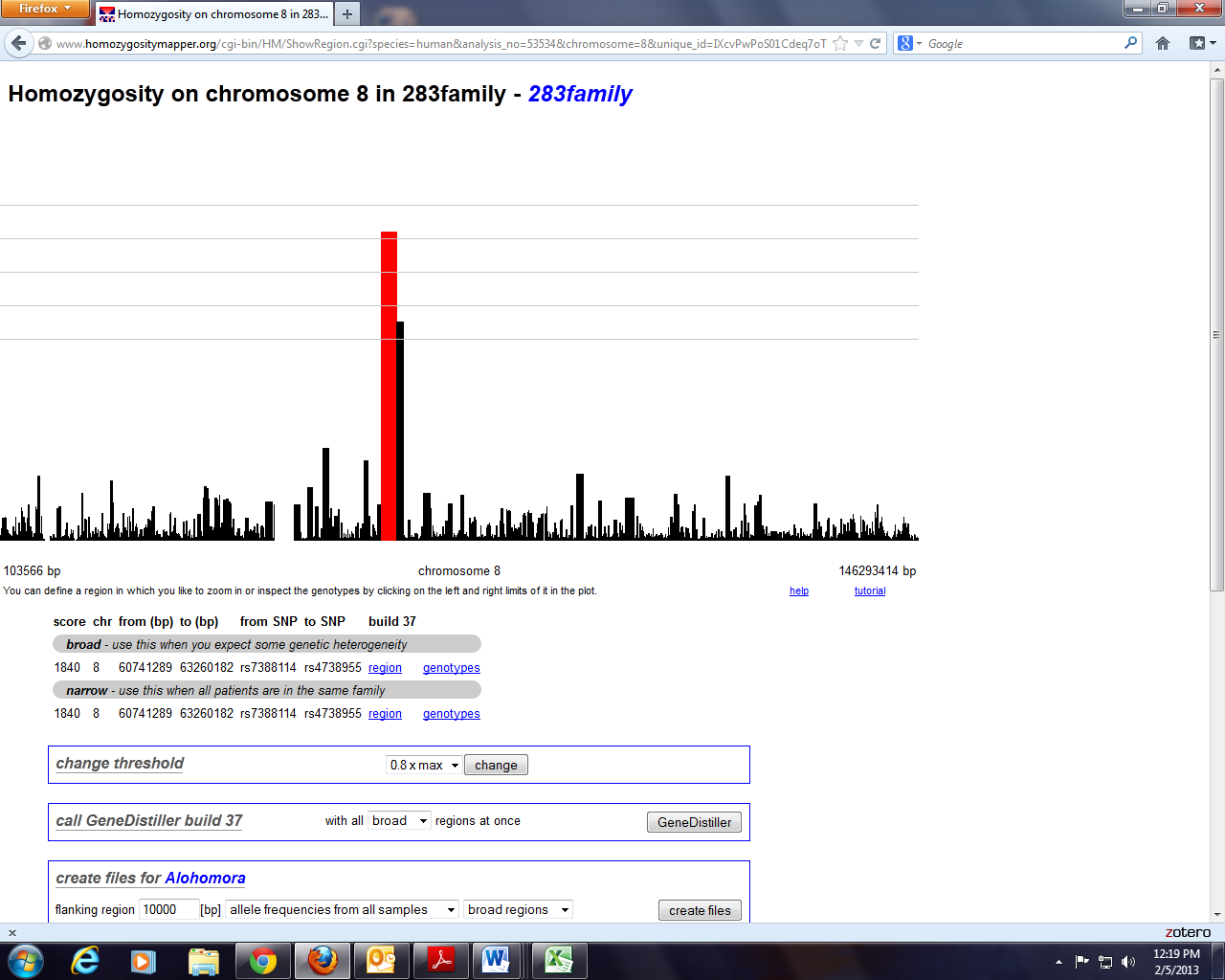


**C)**


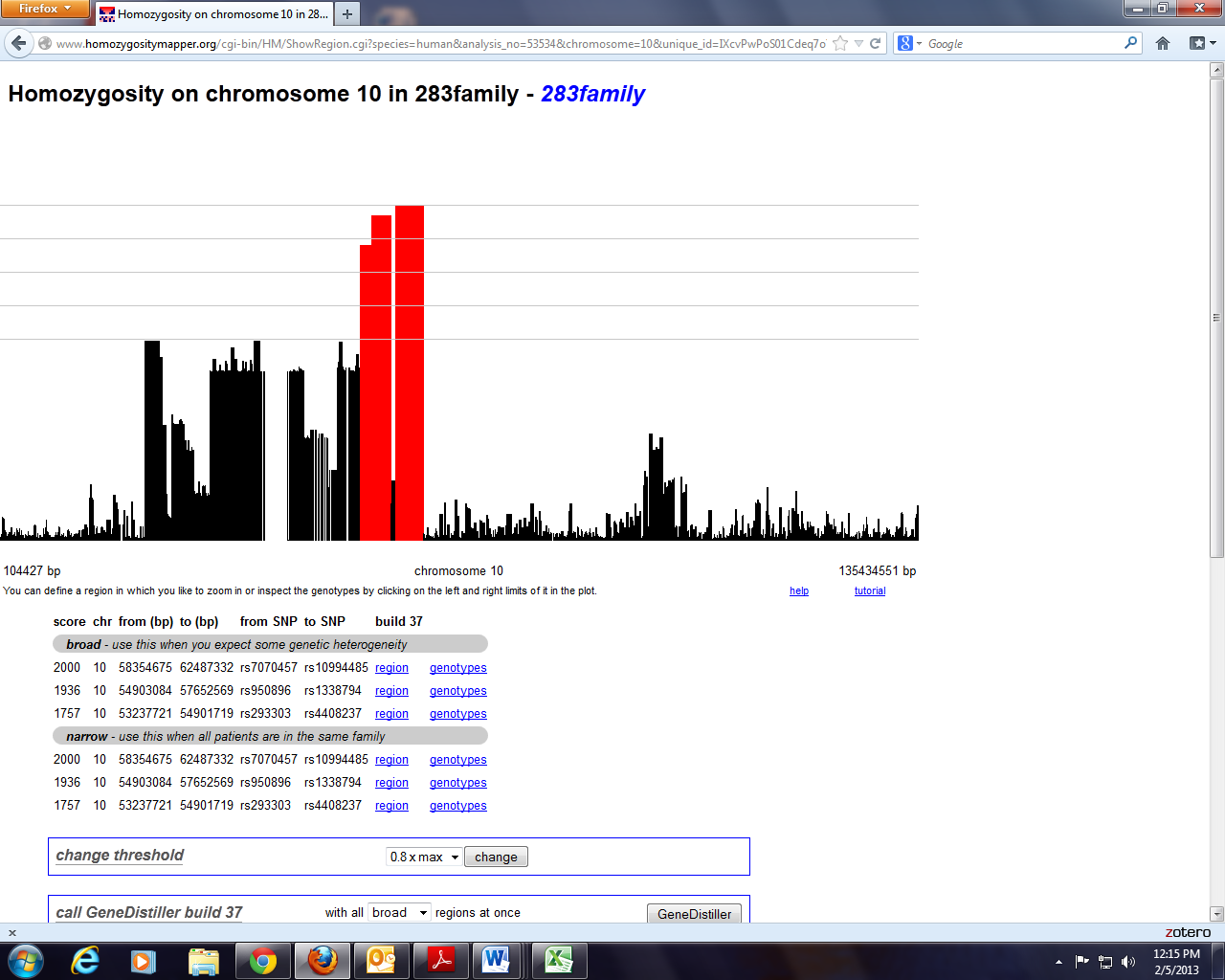

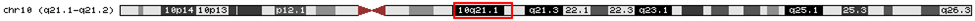

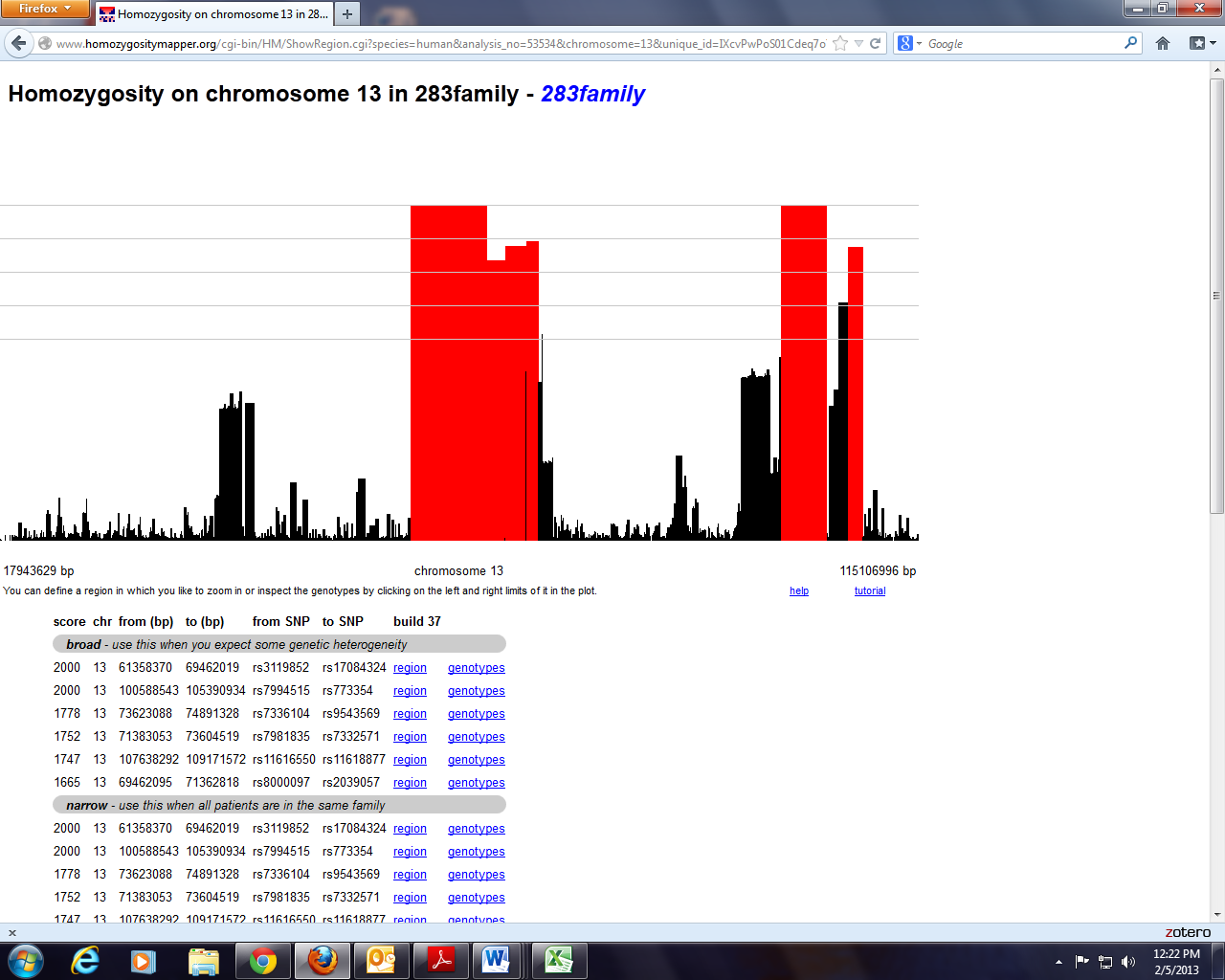

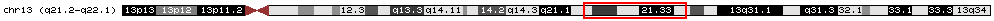

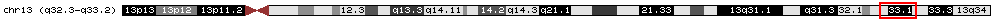

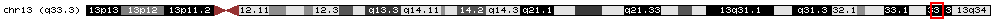


**D)**

**Supplementary Figure S1.** HomozygosityMapper view of the **A)** Genome-wide homozygosity in the studied family (Max homozygosity score: 2000, 868063 markers*),* **B)** Homozygosity on chromosome 8 (q12.1-q12.3), **C)** Homozygosity on chromosome 10 (q21.1-q21.2), **D)** Homozygosity on chromosome 13 (q21.2-q22.1), (q32.3-q33.2) and (q33.3).


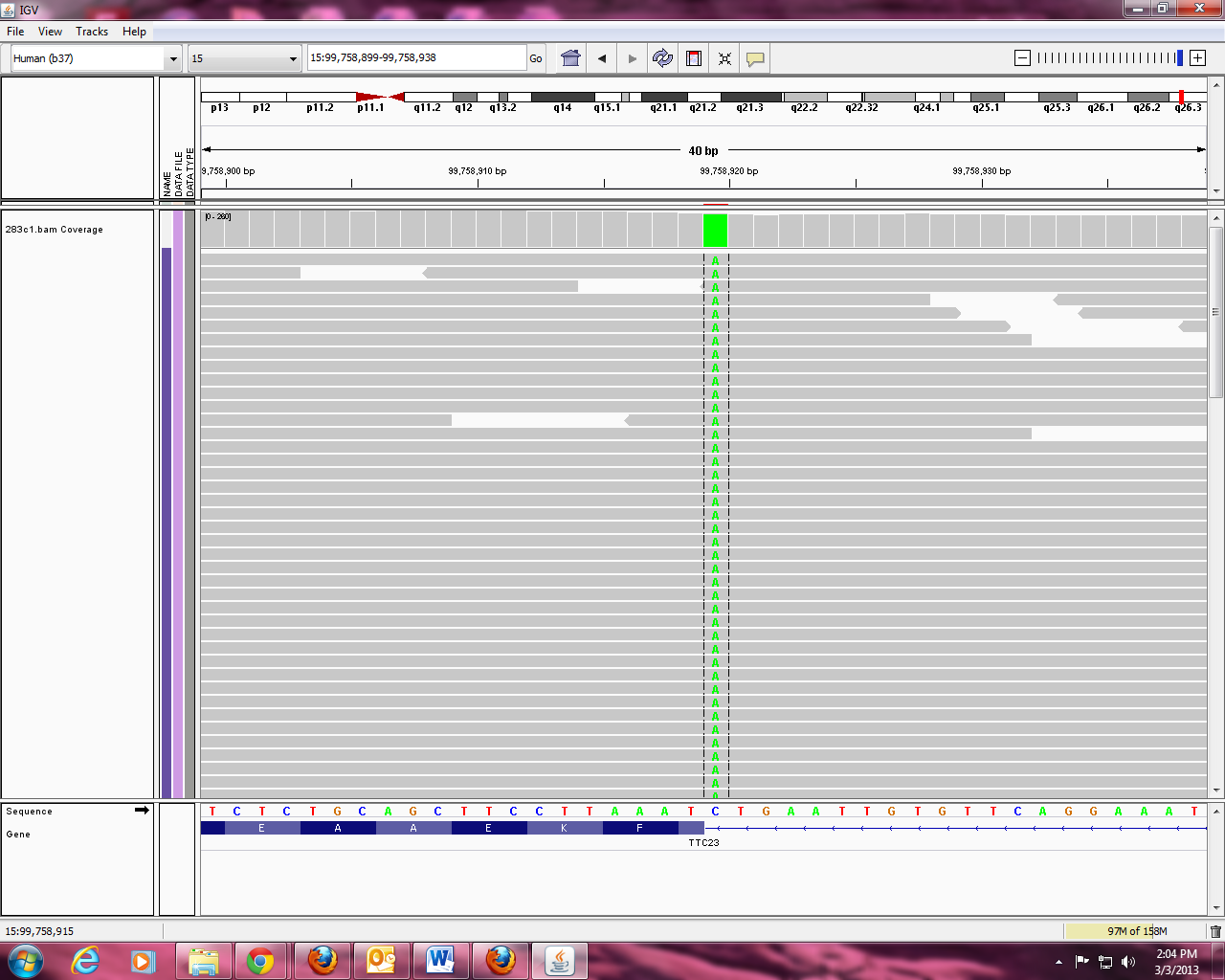


**Supplementary Figure S2.** IGV view of the second homozygous mutation detected by whole exome sequencing on Chr15:99758919C>A in *TTC23* gene. Read depth= 218.


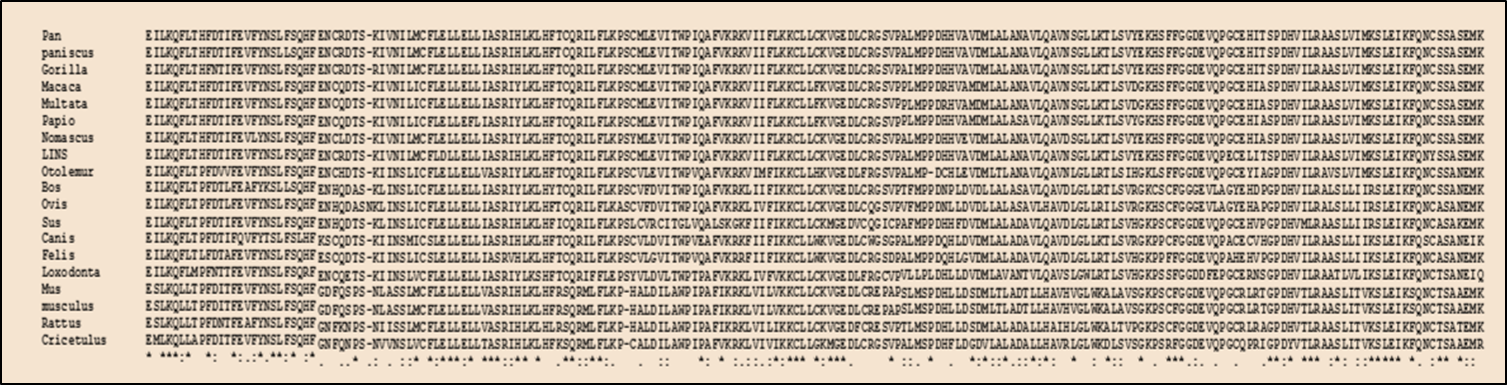


**Supplementary Figure S3.** Conservation across species of the amino acids that are predicted to be deleted from LINS protein in the patients. Alignments adopted from NCBI HomoloGene (http://www.ncbi.nlm.nih.gov/homologene).
